# Using Multiple Microenvironments to Find Similar Ligand-Binding Sites: Application to Kinase Inhibitor Binding

**DOI:** 10.1371/journal.pcbi.1002326

**Published:** 2011-12-29

**Authors:** Tianyun Liu, Russ B. Altman

**Affiliations:** 1 Department of Genetics, Stanford University, Stanford, California, United States of America; 2 Department of Bioengineering, Stanford University, Stanford, California, United States of America; Medical College of Wisconsin, United States of America

## Abstract

The recognition of cryptic small-molecular binding sites in protein structures is important for understanding off-target side effects and for recognizing potential new indications for existing drugs. Current methods focus on the geometry and detailed chemical interactions within putative binding pockets, but may not recognize distant similarities where dynamics or modified interactions allow one ligand to bind apparently divergent binding pockets. In this paper, we introduce an algorithm that seeks similar microenvironments within two binding sites, and assesses overall binding site similarity by the presence of multiple shared microenvironments. The method has relatively weak geometric requirements (to allow for conformational change or dynamics in both the ligand and the pocket) and uses multiple biophysical and biochemical measures to characterize the microenvironments (to allow for diverse modes of ligand binding). We term the algorithm PocketFEATURE, since it focuses on pockets using the FEATURE system for characterizing microenvironments. We validate PocketFEATURE first by showing that it can better discriminate sites that bind similar ligands from those that do not, and by showing that we can recognize FAD-binding sites on a proteome scale with Area Under the Curve (AUC) of 92%. We then apply PocketFEATURE to evolutionarily distant kinases, for which the method recognizes several proven distant relationships, and predicts unexpected shared ligand binding. Using experimental data from ChEMBL and Ambit, we show that at high significance level, 40 kinase pairs are predicted to share ligands. Some of these pairs offer new opportunities for inhibiting two proteins in a single pathway.

## Author Summary

Small molecule drugs may interact with many proteins. Some of these interactions may cause unexpected effects, including side effects or potentially useful therapeutic effects. One way to predict these effects is to analyze the three-dimensional structure of target proteins, and identify new binding sites for small molecule drugs. Several methods have been proposed for predicting new binding sites, relying on geometric and functional complementarity of the sites and the small molecules. In this paper, we report on a new method for identifying novel protein-drug interactions by analyzing the similarity between binding sites in proteins. The method has relatively weak geometric requirements and allows for conformational change or dynamics in both the ligand and protein. Our results show that geometric flexibility is useful for effectively comparing sites. We have applied the method to evolutionarily distant kinases, and find unexpected shared inhibitor binding. Our results may be valuable for drug repurposing in order to find novel uses for existing kinase inhibitors.

## Introduction

Structural biology studies have provided large numbers of high-resolution proteins, often bound to small molecule ligands. The ability to predict additional ligands that will bind these proteins is an exciting opportunity for understanding drug action and repurposing. In some cases, the binding of a small molecule to a protein may explain otherwise unexpected effects of the small molecule, such as side effects of drugs. In other cases, the binding of a small molecule may suggest new uses of existing drugs, based on unexpected affinity to new targets.

Previous methods for predicting the potential binding of small molecules to protein pockets have used evolutionary, structural, biochemical and geometric properties in order to assess pocket similarity, or ligand-pocket complementarity [1], [2], [3], [4], [5]. For example, the method of sequence order-independent profile-profile alignment (SOIPPA) [6] can recognize binding site similarity between the cholesteryl ester transfer protein (CETP) and off-targets, including retinoid X receptor and peroxisome proliferator-activated receptors (PPARs). These new targets may explain adverse drug effects of CETP inhibitors [7]. SOIPPA represents binding sites with a tessellation of C-alpha atoms and characterizes binding sites using geometric similarity potentials. SOIPPA evaluates 3D alignments between binding sites that are enriched for similar angles and distances between residues. It then gauges overall similarity based on geometric criteria, evolutionary and biochemical properties.

Like SOIPPA, other methods for locally comparing binding sites typically have three steps [5]: (1) representation of binding sites, (2) 3D alignment between two sites and (3) evaluation of a similarity metric to the two sites. Searching for the best 3D alignment is the essential step. There are geometric hashing methods (SiteEngine [8] and SiteBase [1]) and methods based on clique detection (SOIPPA [6], CavBase [9] and eF-site [10]). These methods use thresholds to control the similarity of local geometries in both types of methods, but these can be difficult to set. In particular, flexible matching can be critical in achieving high performance [11]. Thornton et al showed that binding sites with similar ligands display greater conformational variability than the corresponding ligand molecules [12]. Thus, using predefined geometric models and thresholds is not optimal. Excessive reliance on crystallographic poses for both the protein and the ligand can miss potential similarities.

We have previously described the FEATURE methods for describing active sites [13]. In the FEATURE representation, a protein site is represented by one or more microenvironments—statistical descriptions of the occurrence of atoms, residues, secondary structures as well as biochemical and biophysical properties in radial shells around a central point [14]. We have shown that the FEATURE representation is useful for describing sites for Ca++ binding [14], Mg++ binding [15], serine protease active sites [13], thioredoxin active sites [16] and others [17]. We have also used it to evaluate the ability of engineered loops to bind ligands [18]. In this work, we reasoned that the interactions between microenvironments in the target protein and chemical fragments in the ligand may drive molecular recognition. Because the conformational arrangement of fragments within flexible ligand molecules can be very different, we allow the corresponding microenvironments to adopt different relative geometries. We develop an algorithm, PocketFEATURE, to match microenvironments within pockets in order to find pockets with potentially similar binding capabilities.

We validate PocketFEATURE by testing its performance on two tasks. First, we test its ability to detect the similarity of binding sites that are known to bind the same (containing adenine-ribose) ligands–a test of sensitivity. Second, we test its ability to detect FAD binding across a large proteome-scale set of proteins–a test of specificity. In both tests, the method shows very strong performance, including an ability to detect similarities missed by other methods.

Kinases play an important role in cell signaling, and can be dysregulated in cancer. Several recently introduced cancer drugs act as ATP analogues and inhibit kinase action [19],[20]. Therefore, the binding capabilities (or binding profiles) of kinases may be useful for discovering novel kinase inhibitors. In particular, kinase inhibitors that bind two or more kinases in the same pathway are attractive because they can more effectively interfere with the pathway, without the need for very high doses or multiple drugs [21], [22]. Kinase binding profiles may also be useful for understanding side effects of drugs that bind multiple proteins [22]. In principle, the ability of an inhibitor to bind multiple kinases may occur despite very low sequence and structure homology. For these reasons, the ability to detect similar binding sites in divergent kinases is potentially valuable.

There are sixteen cancer drugs approved or in advanced development that are known to have multiple targets [22]. All of these drugs target members of the same protein family that regulate the same signaling process. Accordingly, most studies have focused on dissecting the detailed binding preferences of drugs on a relatively small set of kinases that are known to be important. In fact, multi-target drugs that work on proteins within distant families are only rarely reported [23]. Therefore, one of the goals of this work is to discover unrecognized binding similarities between remotely related proteins to increase the repertoire of kinase inhibitor action and utility. Accordingly, we apply PocketFEATURE to predict the similarity in inhibitor-binding profiles between kinases. In particular, we seek similar inhibitor-binding profiles for kinases that are evolutionarily distant.

## Results

### Detecting binding site similarity

**Figure 1:**
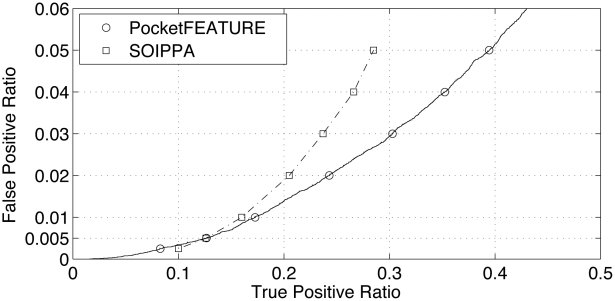
Comparison of the performance of PocketFEATURE to SOIPPA. The benchmark measures the ability to discriminate 30381 pairs of sites that both bind adenine-ribose moieties from 24947 control pairs of sites where one site binds adenine-ribose and the other does not. The AUC for the entire benchmark is 0.85. At the specificity range of 95% to 99.5%, PocketFEATURE outperforms SOIPPA. At 95% specificity, PocketFEATURE identifies about 40% positive pairs, while SOIPPA identifies less than 30%.

We first validated PocketFEATURE's ability to detect binding site similarity. In particular, we tested its ability to recognize pockets that bind similar ligands, and distinguish them from pockets that do not bind these ligands. The benchmark dataset is provided by Bourne group from USCD [6]. The ligands sharing similarity are: ATP, ADP, NAD, FAD, SAH and SAM, all of which contain an adenine-ribose moiety. We compared the performance of PocketFEATURE to SOIPPA, which outperforms other ligand-binding site comparison algorithms in this task [6]. Figure 1 shows PocketFEATURE's ability to recognize 30381 pairs of sites that both bind adenine-ribose moieties, and to recognize the lack of similarity of 24947 control pairs of sites in which one site binds adenine-ribose and the other does not. The AUC for the entire benchmark is 0.85. At specificities of 95% to 99.5%, PocketFEATURE outperforms SOIPPA. At 95% specificity, PocketFEATURE identifies about 40% similar pairs, while SOIPPA identifies less than 30%. Our results demonstrate that PocketFEATURE can identify binding sites with overlapping chemical specificity. Proteins in this test are evolutionarily divergent (<5% of them are from the same SCOP superfamily). Thus, PocketFEATURE can detect site similarity across remote evolutionary relationships.

**Figure 2:**
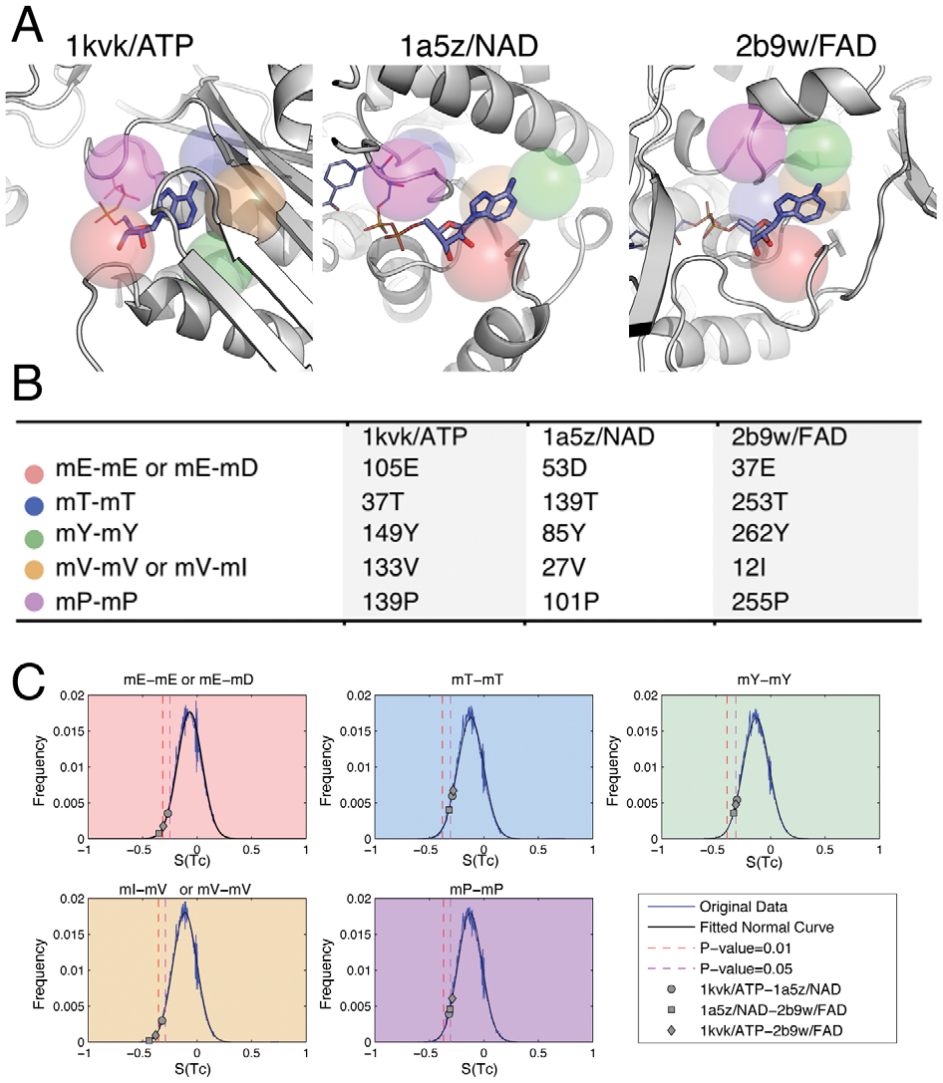
An example illustrating how PocketFEATURE identifies similar sites that bind to ligands with overlapping chemical specificity. We compare an ATP-binding site (1kvk/ATP), an NAD-binding site (1a5z/NAD) and an FAD-binding site (2b9w/FAD). There are five sets of mutual aligned microenvironments between the three sites. (A) 3D structures of the binding site. The five sets of mutual aligned microenvironments are represented as colored spheres: red for microenvironment centered at residue type E(mE) or D (mD), blue for mT, green for mY, orange for mV or mI and purple for mP. The five microenviroments are close to the adenine moiety from ATP, FAD and NAD molecules. The aligned microenvironments display different relative geometries in the three sites. 3D illustrations were generated using PyMOL [37]. (B) Index of center residues for the mutual aligned microenvironments. (C) Significance of alignments. Tc scores between the five sets of mutual aligned microenvironments are mapped to the pre-calculated PDF for a given type of microenvironment pair. The scores between ATP-binding and NAD-binding sites are marked using diamonds, those between ATP-binding and FAD-binding using circles, and those between NAD-binding and FAD-binding using squares. For each of the five sets, the S(Tc) scores fall within the p-value cutoff of 0.05.

Figure 2 shows an ATP-binding site (1kvk), an NAD-binding site (1a5z) and an FAD-binding site (2b9w). There are five corresponding microenvironments in each protein (spheres are shown in Figure 2A and the types of microenvironments are listed in Figure 2B). These five microenviroments are in proximity to the adenine moiety from ATP, NAD and FAD molecules. It is important to note that the five microenvironments adopt different relative geometries in these three sample sites while coordinating the ligands. We consider the five microenvironments to constitute modules capable of recognizing adenine-ribose moieties contained within diverse ligand molecules.

The S(Tc) score captures the similarity of a pair of FEATURE microenvironments. To evaluate the significance of alignments between microenvironments, we calculated the probability distribution function (PDF) of microenvironment similarity score S(Tc) using a non-redundant dataset of 3D structures in PDB. Given each set of the mutual alignment between ATP, NAD and FAD binding sites, Figure 2C maps the three S(Tc) scores to the corresponding microenvironment types. The alignments of high significance form the basis for recognizing similarity between binding sites detected by PocketFEATURE.

### Detecting FAD-binding sites in a large druggable database

**Figure 3:**
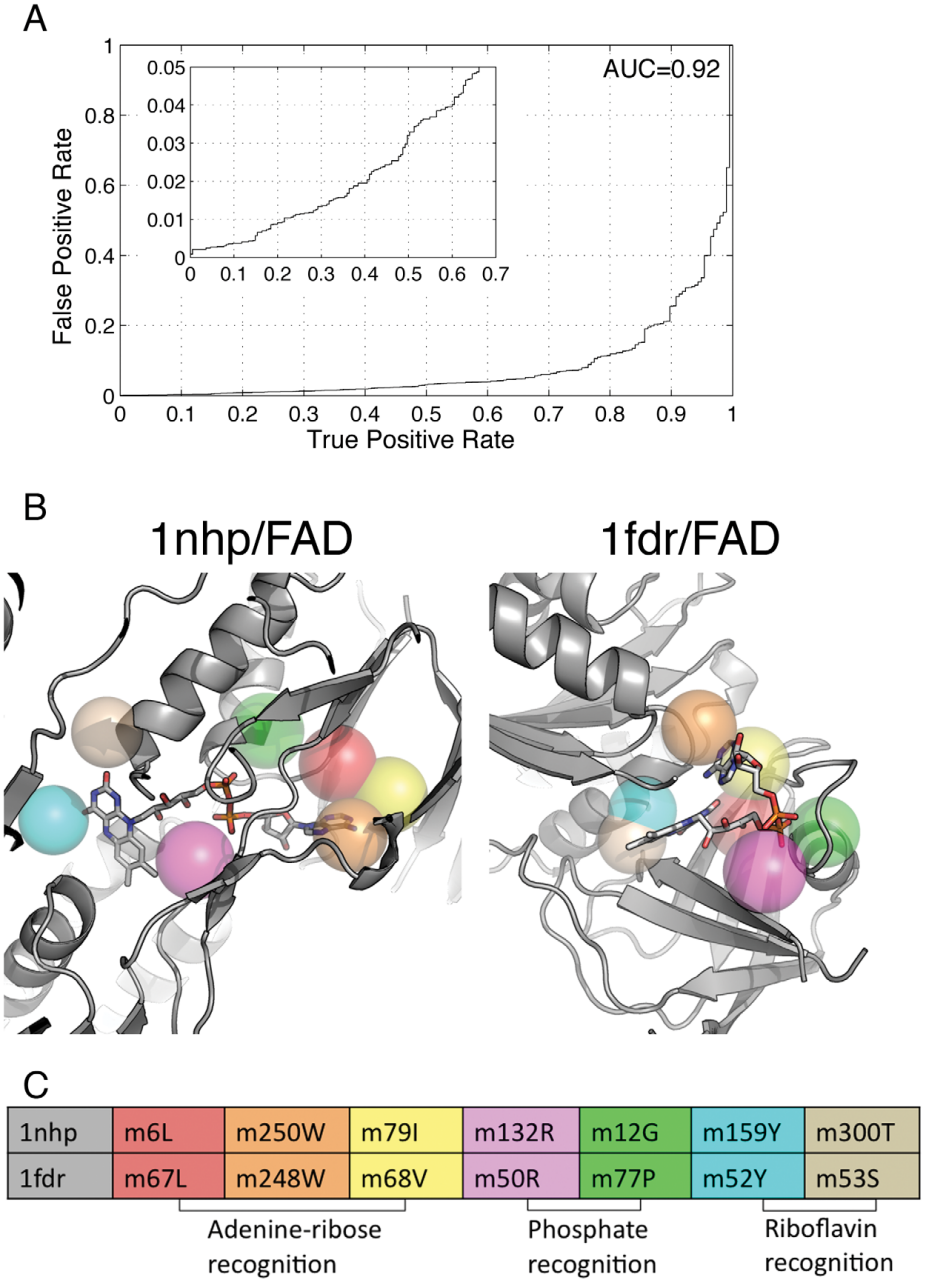
Identification of FAD-binding sites in a large druggable database. (A) The overall performance with an AUC value of 0.92 At 95% specificity, PocketFEATURE identified about 65% sites that are known to bind FAD. At 80% specificity, PocketFEATURE identified nearly 90% sites correctly. (B) Microenvironment alignments between two FAD-binding sites. Two sites adapt to two different ligand conformations, an elongated (1nhp) and a bent butterfly conformation (1fdr). The relative geometries arrangements of aligned microenvironments are different. (C) An illustration of the corresponding positioning of aligned microenvironments and FAD molecules. The aligned microenvironments m6L/1nhp-m67L/1fdr (red), m250W/1nhp-m248W/1fdr (orange) and m79I/1nhp-m68V/1fdr (yellow) are near the adenine moiety of FAD molecules in 1nhp and 1fdr, respectively. Near phosphate chemical groups, aligned m132R/1nhp-m77P/1fdr (pink) and m12G/1nhp-m77P/1fdr (green) are observed. Another two sets of microenvironments m159Y/1nhp-m52Y/1fdr (cyan) and m300T/1nhp-m53S/1fdr (wheat) are found close to flavin groups.

The previous test demonstrated that PocketFEATURE can *sensitively* detect similarities between adenine-ribose containing pockets. We tested PocketFEATURE's ability to *specifically* recognize FAD-binding sites on a structural proteome scale. The Dataset-6958 is derived from an annotated database of “druggable binding sites” from PDB called scPDB (see Method section). It includes a total of 6709 non-FAD-binding proteins and 249 FAD-binding proteins, from 43 EC-families. Using a single arbitrarily selected FAD-binding site (1nhp/FAD) as a query structure, we searched the database for all other FAD-binding sites from Dataset-6958 by pairwise comparison. Figure 3A shows the overall performance with an AUC value of 0.92. At 95% specificity, PocketFEATURE identifies about 65% sites that are known to bind FAD. At 80% specificity, PocketFEATURE identifies nearly 90% sites correctly.

Of note, FAD binds proteins in two general conformations [24]: the (1) elongated and the (2) bent butterfly conformation (Figure 3B). In the bent conformation (1nhp in Figure 3B), the AMP portion is folded back, placing the adenine and isoalloxazine rings in close proximity, whereas in the elongated conformation (1fdr in Figure 3B) the adenine ring is distant from the isoalloxazine ring. Remarkably, PocketFEATURE can detect bent butterfly FAD sites based on an elongated FAD query structure. Conversely, using a binding site with a bent butterfly FAD as a query structure, PocketFEATURE can detect elongated FAD binding sties (Supplementary Material Figure S1).

Figure 3B and 3C show the microenvironment alignments detected by PocketFEATURE. (See Method section, A microenvironment is named using the following convention: “m” followed by “residue index” and “residue type”, upon which the microenvironment is centered. PDB identifier or gene name is tagged when necessary.) The aligned microenvironments m6L1nhp-m67L/1fdr (red), m250W/1nhp-m248W/1fdr (orange) and m79I/1nhp-m68V/1fdr (yellow) are near the adenine moiety of FAD in 1nhp and 1fdr, respectively. Aligned microenvironments m132R/1nhp-m77P/1fdr (pink) and m12G/1nhp-m77P/1fdr (green) are adjacent to phosphate chemical groups. Another two sets of microenvironments m159Y/1nhp-m52Y/1fdr (cyan) and m300T/1nhp-m53S/1fdr (wheat) are found near flavin groups. As might be expected, the different conformations of FAD molecules in the structures 1nhp and 1fdr lead to markedly different geometric arrangements of these aligned microenvironments. However, PocketFEATURE identifies these alignments with high significance, demonstrating the robustness of the algorithm.

### Other related benchmarks

We have performed three independent experiments to test PocketFEATURE's ability to *specifically* recognize non-adenine ligand binding sites. First, using a typical steroid-binding site (1A28 bound with progesterone) to detect all other steroid-binding sites (total 83 sites) from Dataset-6985 (Supplementary Material Figure S2). The overall AUC is 0.826. Second, we compared PocketFEATURE to a 3D shape descriptor using real spherical harmonic expansion coefficients [25], [26]. Using their published datasets (10 sites for ATP, 10 for NAD, 10 for heme and 10 for steroids), PocketFEATURE successfully clusters the four types of sites and compares favorably with the real spherical harmonic shape descriptor (Supplementary Material Figure S3). Third, we applied PocketFEATURE to predicted off-targets for Torcetrapib from a non-redundant subset of PDB for 1200 human proteins. Supplementary material Table S1 lists top ranked off-targets predictions. Xie et al [7] published a panel of 20 off-targets for CETP inhibitors (specifically Torcetrapib) predicted by SOIPPA. These predictions have been refined and validated by docking methods and critical human curation. Comparing the 20 published off-targets by SOIPPA and the 36 predictions by PocketFEATURE, we find that seven are the same. These three tests show that PocketFEATURE effectively recognizes non-adenine ligand binding sites.

The Similarity Ensemble Approach (SEA) is a method for calculating chemoinformatics similarities between drug sets [27]. Given two binding sites, we incorporate the similarity scores between experimentally observed ligand molecules calculated by SEA into the PocketFEATURE similarity score between the binding sites. Preliminary results suggest that combining ligand chemoinformatics and target physiochemical properties leads to strong signals for similarity detection (Supplementary Material Figure S4).

### Predicting inhibitor-binding for distantly related kinases

Having established reasonable sensitivity and specificity of PocketFEATURE, we studied ATP-binding sites in kinases, to find new targets for these inhibitors. We specifically targeted kinase proteins that are distantly related, with little detectable sequence or structural similarity, because most previous work has focused on the binding properties of closely related kinases.

**Figure 4:**
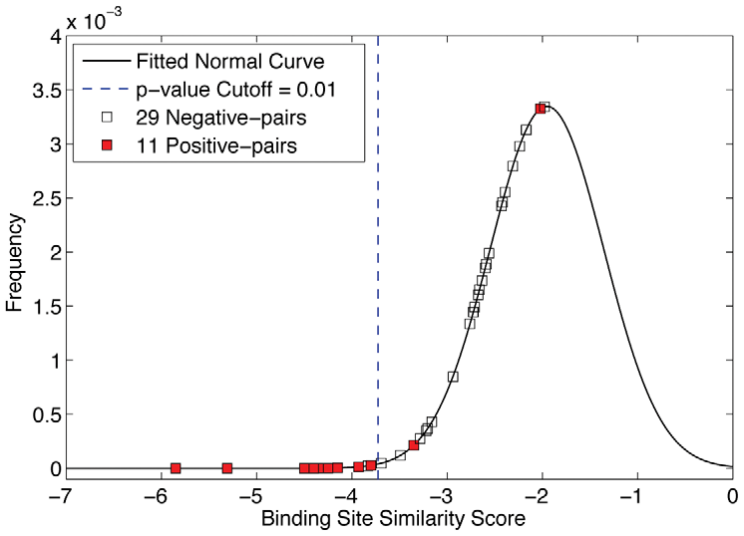
Predicting overlapping of inhibitor-binding profiles between kinases. The binding site similarity scores of 6058 pairs of distant kinases were fitted into normal distribution. The more negative the score, the higher level of similarity between two sites is predicted. The blue dotted line is the p-value cutoff of 0.01 for highly ranked predictions (Table 1). Of the 6058 pairs, there are 40 pairs of which experimental data are available to both kinases. Out of the 40 pairs, 11 pairs have experimental results suggesting overlap in inhibitor binding – they are “positive-pairs”. The remaining 29 pairs are “negative-pairs” that do not share ligands. The significance of the separation of positive-pairs and negative-pairs in this ranking, as evaluated by a hyper-geometric distribution has a *p-value* of 4.5e-18.

We calculated the binding site similarity scores of the 6058 pairs sites in distantly related kinases using PocketFEATURE. Figure 4 plots the PDF of the scores for the 6058 pairs. A small p-value suggests an increased likelihood of high binding site similarity for the pair of proteins evaluated.

CHEMBL is a manually curated chemical database of bioactive molecules with drug-like properties [28]. Our local database downloaded from CHEMBL includes binding assays between a total of 3199 protein domains and 541,137 compounds. Similarly, the Ambit panel provides high-throughput kinase selectivity profiling of 317 kinases against 37 known kinase inhibitors [29]. Of the 6058 pairs we evaluated, there are 40 pairs for which experimental data (from CHEMBL or Ambit) are available for both kinases. Of these pairs, 11 had experimental results suggesting high overlap in inhibitor binding—they are “positive-pairs”. When we rank order the 40 pairs based on their scores, nine of the 11 positive-pairs rank first through eighth, and tenth (Figure 4). The binding site similarity scores of negative-pairs range from −1.9 to −3.6—quite separate from the positives. The significance of the separation of positive-pairs and negative-pairs in this ranking, as evaluated by a hyper-geometric distribution has a *p-value* of 4.5e-18.

Using a p-value cutoff <0.01 from the PDF (binding site similarity score cutoff of −3.76 in Figure 4), 50 pairs of kinases are predicted to have inhibiting-profile overlapping. Nine pairs have experimental evidence suggesting overlap in inhibitor binding- these are true positives (Table 1). One pair is considered false positive because although the two kinases are tested in CHEMBL, they do not share ligands using our relatively stringent cutoff (Table 2). The other 40 pairs with high apparent similarity are novel predictions (Table 3). Experimental evidence for these kinases pairs is not available in CHEMBL or Ambit.

**Table 1:** True positives predictions.

EC group		PDB ID		Gene name		PID	Binding sitesimilarity	Representativeinhibitor
2.7.10.2	2.7.11.22	2src	3blq	SRC	CCNT1	10. 24	−5.85	AST-487
2.7.10.1	2.7.11.26	2hen	1j1c	Ephb2	GSK3B	25.63	−5.31	CHEMBL247067
2.7.10.1	2.7.11.26	1jqh	1j1c	IGF1R	GSK3B	27.86	−4.34	CHEMBL215803
2.7.10.1	2.7.11.22	2hen	2cch	Ephb2	CDK2	19.98	−4.30	AST-487
2.7.1.153	2.7.10.2	1e8x	2src	PIK3CG	SRC	18.26	−4.28	PP121
2.7.10.1	2.7.11.22	1jqh	2cch	IGF1R	CDK2	23.94	−4.25	BMS-536924
2.7.10.1	2.7.11.24	2hen	1cm8	Ephb2	MAPK12	26.61	−4.15	AST-487
2.7.10.1	2.7.11.24	1jqh	1cm8	IGF1R	MAPK12	29.36	−3.83	CHEMBL215803
2.7.1.153	2.7.11.1	1e8x	2pvr	PIK3CG	CSNK2A1	23.17	−3.80	CHEMBL379156

Using a p-value cutoff of 0.01, 50 pairs of kinases are predicted to share inhibitors. Nine pairs have experimental evidence suggesting overlap in inhibitor binding- these are **true positives**. The first two columns are EC groups. Kinases in one pair are from different EC groups. The PDB IDs and gene names are listed in column three to row six. The seventh column lists the percentage identity (PID) of structural alignment between two kinases in a pair. The eighth column shows the binding site similarity score of the pair. The more negative the score is, the higher the similarity level between two sites is predicted.

**Table 2 pcbi-1002326-t002:** False positive predictions.

EC group		PDB ID		Gene name		PID	Binding site similarity
2.7.1.153	2.7.12.2	1e8x	1s9j	PIK3CG	MAP2K1	28.37	−3.88

One pair is considered false positive because both kinases are linked to at least one set of experimental data from CHEMBL, but they do not share ligands using our stringent standards.

**Table 3:** Novel predictions of high similarity.

EC group		PDB ID		Gene name		PID	Binding sitesimilarity
2.7.11.2	2.7.13.3	2e0a	3d36	PDK4	spo0ANE	28.54	−6.6061
2.7.11.2	2.7.13.3	1jm6	3d36	Pdk2	spo0ANE	28.75	−5.4505
2.7.1.95	2.7.11.1	1j7u	1zp9	aphA	rio1	12.55	−5.4243
2.7.11.1	2.7.13.3	1th8	2c2a	spoIIAB	TM_0853	12.5	−5.2046
2.7.1.100	2.7.11.1	2pyw	1csn	At1g49820	cki1	28.33	−4.9383
2.7.1.100	2.7.11.1	2olc	1zp9	mtnK	rio1	10.24	−4.6189
2.7.1.100	2.7.11.1	2pyw	1u5r	At1g49820	Taok2	10.71	−4.475
2.7.1.100	2.7.11.24	2olc	1cm8	mtnK	MAPK12	10.55	−4.4283
2.7.1.153	2.7.11.1	1e8x	1zyd	PIK3CG	GCN2	11.09	−4.4036
2.7.10.2	2.7.11.1	2ijm	1zp9	PTK2	rio1	25.91	−4.4022
2.7.10.1	2.7.11.1	1mqb	1zp9	EPHA2	rio1	25.76	−4.3987
2.7.1.100	2.7.11.1	2olc	1zyd	mtnK	GCN2	13.19	−4.3499
2.7.1.100	2.7.11.1	2pyw	3e7e	At1g49820	BUB1	28.83	−4.3048
2.7.1.153	2.7.11.1	1e8x	1lp4	PIK3CG	ACK2	23.24	−4.2874
2.7.1.100	2.7.11.1	2olc	3e7e	mtnK	BUB1	25.83	−4.2698
2.7.1.95	2.7.11.1	1j7u	3e7e	aphA	BUB1	7.21	−4.2628
2.7.1.100	2.7.11.26	2pyw	1j1c	At1g49820	GSK3B	12.26	−4.1925
2.7.1.100	2.7.12.2	2pyw	1s9j	At1g49820	MAP2K1	27.68	−4.1752
2.7.10.1	2.7.11.1	2hen	1q97	Ephb2	SKY1	21.35	−4.1734
2.7.1.100	2.7.10.2	2olc	2ozo	mtnK	ZAP70	12.66	−4.1549
2.7.1.36	2.7.12.2	1kvk	1s9j	Mvk	MAP2K1	11.07	−4.1171
2.7.10.1	2.7.11.1	1jqh	2vwi	IGF1R	OXSR1	24.17	−4.1001
2.7.1.100	2.7.11.24	2pyw	1cm8	At1g49820	MAPK12	10.86	−4.0862
2.7.10.1	2.7.11.1	1pkg	1zp9	KIT	rio1	24.51	−4.077
2.7.1.100	2.7.11.1	2pyw	1o6l	At1g49820	AKT2	29.75	−4.0536
2.7.1.100	2.7.10.2	2pyw	2src	At1g49820	SRC	20.49	−4.0529
2.7.1.-	2.7.11.1	2a19	1zp9	SUI2	rio1	20.29	−4.0507
2.7.1.100	2.7.11.1	2pyw	1zyd	At1g49820	GCN2	13.19	−4.0412
2.7.10.1	2.7.11.1	2hen	1zp9	Ephb2	rio1	14.78	−4.0407
2.7.10.2	2.7.11.1	2src	1zp9	SRC	rio1	24.28	−4.0122
2.7.1.95	2.7.11.1	1j7u	1tqp	aphA	rio2	14.83	−4.0018
2.7.1.100	2.7.11.1	2olc	1u5r	mtnK	Taok2	12.82	−3.9498
2.7.1.95	2.7.11.1	1j7u	1u5r	aphA	Taok2	19.01	−3.9436
2.7.1.100	2.7.10.1	2pyw	1pkg	At1g49820	KIT	17.11	−3.9048
2.7.11.4	2.7.4.22	1gkz	2bri	Bckdk	pyrH	11.08	−3.8977
2.7.1.100	2.7.12.2	2olc	1s9j	mtnK	MAP2K1	27.68	−3.8804
2.7.1.95	2.7.11.24	1j7u	1cm8	aphA	MAPK12	11.79	−3.8187
2.7.1.36	2.7.10.1	1kvk	2qoc	Mvk	EPHA3	10.6	−3.8101
2.7.1.100	2.7.10.2	2pyw	2ozo	At1g49820	ZAP70	15.23	−3.8093
2.7.1.144	2.7.11.1	2f02	1u5r	lacC	Taok2	7.63	−3.8036

In the top 50 ranked predictions, there are 40 pairs of **novel predictions** that experimental evidence for one for both kinases is not available in CHEMBL or Ambit.

## Discussion

We have presented a new algorithm for detecting ligand-binding site similarity, tested it on (1) the recognition of adenine-ribose binding ligands and (2) the recognition of FAD binding sites. We then applied it to the problem of predicting cross binding of ATP analogues inhibitors of kinases.

### Summary of PocketFEATURE method

Our method works for two reasons. First, we employ a novel microenviroment-based representation and scoring system for comparing pockets that captures the physical and chemical properties in the binding pocket, and second, we do not impose rigid geometric matching criteria on the microenvironments within the pocket. The resulting method accurately recognizes similar microenvironments, and identifies combinations of microenvironments that can interact with fragments within ligand molecules.

#### Representation and similarity measure between individual microenvironments

Microenvironments are represented using the FEATURE representation that captures physiochemical properties of a local subsite. The raw Tc score measures similarity between two microenvironments. However, it is difficult to compare Tc scores across pairs of microenvironments because the background similarities between different pairs are not the same. Therefore, it is necessary to normalize the Tc scores. We normalize the scores by creating a background distribution for each pair type (See Method). The normalized score S(Tc) is negative, decreases monotonically with increasing Tc, and changing most rapidly for Tc>Tc_0_. Therefore, our method seeks microenvironment-pairs that have high similarity, given the expected background score distribution.

We aligned microenvironments from an ATP, FAD and an NAD binding site (Figure 2). Microenvironment similarity score S(Tc) of aligned ones are outliers within the PDF (Figure 2C). The aligned microenvironments constitute functional modules for ligand recognition (Figure 2A). That is, particular microenviroments are associated with the recognition of particular molecular fragments with ligands. A better (more negative) S(Tc) similarity captures this shared recognition role in different binding sites.

#### Geometric flexible matching between microenvironments

The combined interactions between multiple microenvironments in a target protein and molecular fragments within its ligand molecule drive molecular recognition. Because fragments can adopt different poses within a ligand molecule, the FEATURE based microenvironments in the target protein can adopt different relative geometries, as shown in Figure 3C. Some methods use geometric constraints to control local arrangements of functional modules. PocketFEATURE has relatively weak geometric requirements (only that the matching microenvironments be present within the pocket of interest). As a result, the number of possible alignments between pockets is increased, and PocketFEATURE can recognize site that bind similar ligands in different poses.

Our results with FAD-binding sites illustrate the value of geometric flexibility; FAD contains highly flexible regions between flavin and adenine. The FAD conformation and orientation varies widely across different protein families. By using PocketFEATURE, microenvironments corresponding to the same fragments within FAD are recognized even though these microenvironments adopt different local geometries according to their ligand poses (Figure 3C). These results demonstrate the value of allowing microenvironments to adopt variable orientations within pockets.

We have also assessed whether the microenvironment alignments identified by PocketFEATURE correspond to specific recognition of ligand chemical substructures. The average number of microenvironments between two ATP sites is six; while that of ADP is six, that of NAD is ten and that of FAD is twelve. The common aligned microenvironments of high frequencies for these four types of binding sites are: mD, mE, mR, mK, mS and mT, all of which are in close proximity to the common sub-fragments contained within ADP, ATP, FAD and NAD: the adenine, ribose and phosphate. Thus, these microenvironments have specific roles in recognizing particular fragments within the overall molecule. Furthermore, the additional aligned microenvironments observed in FAD and NAD sites are mW and mP, which “recognize” the phosphate, flavin or nicotinamide moieties.

In summary, PocketFEATURE is more sensitive than other state-of-the-art methods. It is suitable for application on a genome scale.

PocketFEATURE defines sites based on position of known ligand binding. For apo structures and uncharacterized sites, we can also use published patch-searching algorithms, such as CONCAVITY [30] and PocketPicker [2] to define sites (on-going projects).

### Application of PocketFEATURE to drug discovery

Current computational studies on comparing kinase inhibitor binding sites often focus on known drug targets Tyrosine kinase (TK family, EC 2.7.10) and Serine/Threonine kinases ISTE family (EC 2.7.11). Potential similarity between divergent kinases has not been systematically explored either computationally or experimentally. For example, of the 6058 pairs of divergent kinases we compared in this work, only 40 pairs have experimental data from CHEMBL and Ambit. The literature is biased towards a few well-validated kinases. Some inhibitors may appear to be more promiscuous simply because they have been profiled more systematically.

As shown in Figure 4, PocketFEATURE identifies similar ligand binding sites across distantly related kinases. The surprisingly accurate predictions (Table 1) for those that have been tested make the remaining untested predictions (Table 3) with high similarity scores particular interesting.

**Figure 5:**
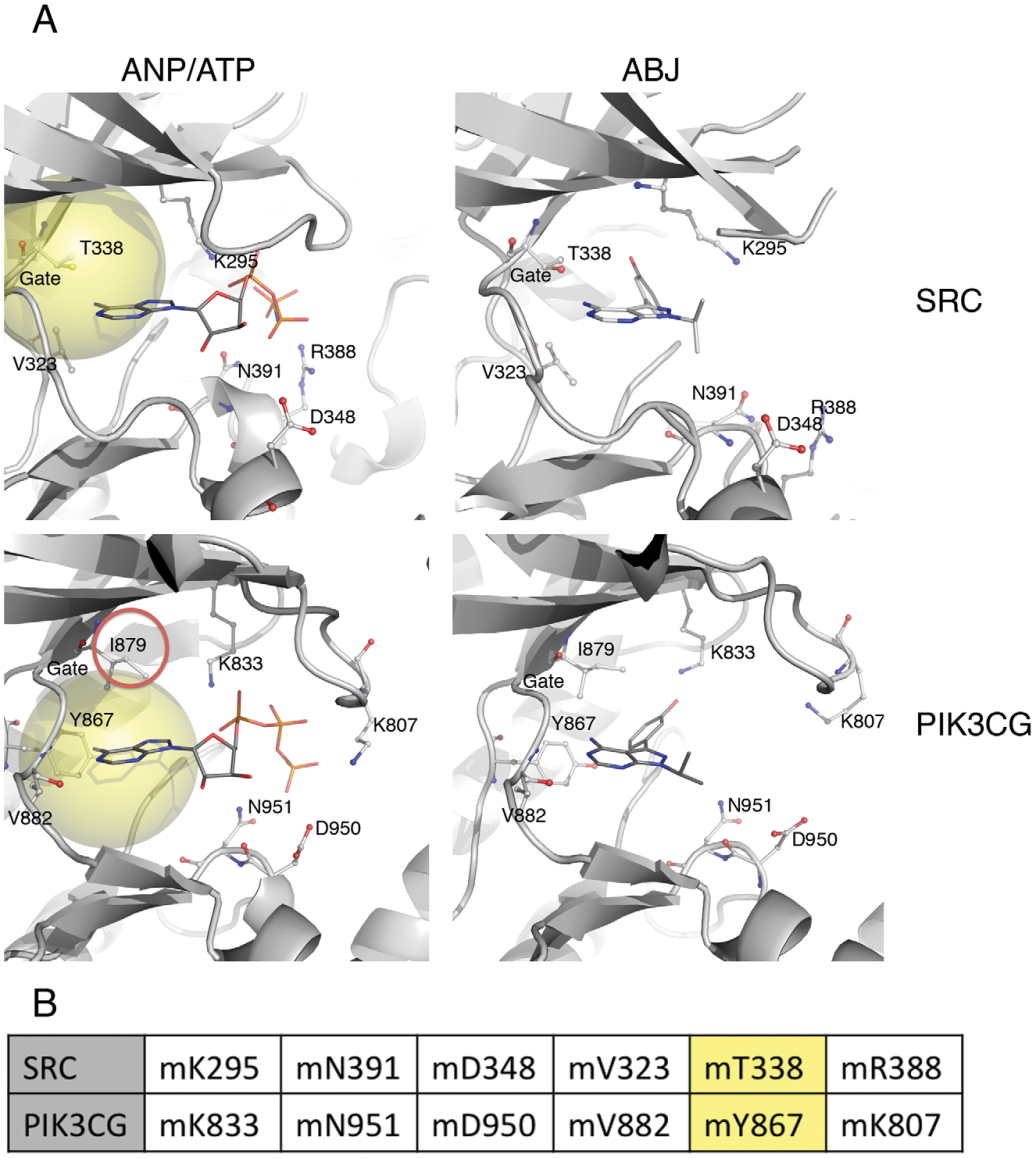
An example of validated positive predictions. SRC (a Tyrosine kinase) and PIK3CG (a lipid kinase) bind to a same series of inhibitors (PP121 and its derivatives). Four related structures are available in PDB. The first row is structures of SRC bound with ANP (PDB ID: 2src) and a drug-like inhibitor (PDB ID is 3en7 and ligand PDB code is ABJ). The second row is structures of PIK3CG bound with ATP (PDB ID is1e8x) and ABJ (PDB ID is 2v4l). Between binding sites of 2src (SRC/ANP) and 1e8x (PIK3CG/ATP), PocketFEATURE aligned six pairs of microenvironments. At the position near the typical “gate” of an ATP-binding site, PocketFEATURE aligned mT338/SRC to mY867/PIK3CG (light yellow sphere). In SRC structures, the gatekeeper residue T338 enables interactions between the deep hydrophobic pocket and ligands. The original experimental study suggests the residue analogous to the gatekeeper in PIK3CG is I879 (red circle), which is the nearest microenvironment to mY867/PIK3CG. (A). 3D illustration of binding sites in SRC and PIK3CG (B). Aligned microenvironments.

Some of our predictions rediscover combinations of divergent kinases for multi-targeted drug design. One highly ranked prediction is the pair SRC (a Tyrosine kinase) and PIK3CG (a lipid kinase in PI3K family), which are evolutionarily distant (PID<10%). Both kinases have been observed to bind to a similar series of inhibitors (PP121 and its derivatives) [23]. The PocketFEATURE binding site similarity score (−4.26) ranks high —20th out of 6058 pairs. PocketFEATURE identifies six key microenvironments (Figure 5). One alignment matches mT338/SRC to mY867/PIK3CG, both of which reside near the typical “gate” of an ATP-binding site. The gatekeeper residue enables interactions between the deep hydrophobic pocket and ligands (adenine in ATP or pyrazolopyrimidine in inhibitor ABJ). In SRC structures, residue T338 is designated as the gatekeeper and in PIK3CG structures, residue I879 [23]. As PocketFEATURE does not seek alignments between polar and hydrophobic residues, it does not find a match between mT338/SRC and mI879/PIK3CG. Instead, in the PIK3CG structure, the proximal mY867/PIK3CG is aligned to mT338/SRC. Thus, PocketFEATURE detects similar binding sites from these two distant kinases. SRC activates the lipid kinases of the PI3K family, a central regulator of cell growth. Molecules that target both SRC and PIK3CG may have potent antitumor activity.

PIK3CG is also predicted to share ligands with other kinases, including CSNK21A, MAP2K1, GCN2 and ACK2 (Table 1–3). Of these, CSNK21A encodes a Casein kinase that is involved in Wnt signaling pathway. Casein kinase inhibitors are considered potent anti-cancer drug candidates [31]. It is possible that drugs inhibiting both CSNK21A and PIK3CG may have synergistic anti-tumor activity. ACK2 is a homolog of CSNK21A (PID>90%). The binding site similarity score between PIK3CG and ACK2 ranks highly—18th out of 6058 pairs.

In addition to its similarity to CSNK21A, the pocket in PIK3CG is very similar to that in MAP2K1. These proteins do not share known ligands using our stringent interpretation of CHEMBL results (see Method). However, this apparent false positive deserves further scrutiny. MAP2K1 encodes an essential kinase in mitogen-activated protein (MAP) kinase signal transduction pathway. Activation of MAP kinase pathway plays important roles in the metastasis of pancreatic cancer. Specific inhibitors have been developed to inhibit oncogenic pathways. However, activation of PI3K pathway in response to MAP2K1 inhibition through a negative feed back loop limits the efficacy [32]. The high similarity between PIK3CG and MAP2K1 by PocketFEATURE suggest the possibility of inhibitors that target both kinases.

Other highly ranked novel predictions have some implications for side effects and drug repurposing. In Table 3, there are three pairs of mammalian: {Mvk-MAP2K1}, {Mvk-EPHA3} and {1GF1R-OXSR1}. Of these kinases, MAP2K1, OXSR1 and EPHA3 are implicated in cancer. Further evaluation of these pairs may be warranted. Most of other pairs in Table 3 are combinations of one kinase from human (or other mammalian) and one from plant (or bacteria). It is intriguing to consider that inhibitors for plant or bacterial kinases may be useful inhibitors of mammalian proteins.

## Methods

### FEATURE microenvironments

Given a functional center of a residue, we use the term “microenvironment” to refer to the local, spherical region in the protein structure that may encompass residues discontinuous in sequence and structure. Specifically, we use the FEATURE system to calculate a set of 80 physicochemical properties (Table S2) [14] collected over six concentric spherical shells (total 480 properties = 80 properties×6 shells) centered on the predefined functional center (Table S3) [33]. The total radius of the microenvironment is 7.5 Angstroms. A microenvironment is named using the following convention: “m” followed by “residue index” and “residue type”, upon which the microenvironment is centered. PDB identifier or gene name is tagged when necessary. For example m6L/1nhp represents the microenvironment centered on the functional center of the sixth residue in 1nhp, which is leucine (L). A complete description of FEATURE can be found in the original publication.

### Similarity measure between two FEATURE microenvironments

Given a pair of FEATURE microenvironments (A and B) derived from two different sites, we calculate an adjusted Tanimoto coefficient based on the presence/absence of similar properties. We compute a single standard deviation (STD) for each of the 480 properties across a random set of FEATURE microenvironments (see section “Background calculation). Two microenvironments have a “similar property” if they differ by less than one STD for the given property. Given A and B, c is the number of “similar properties”; a and b are the numbers of non-zero properties in A and B, respectively; the denominator is the total number of unique properties that are non-zero in A or B or both (a+b−c); then the Tanimoto similarity is as follows: 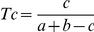.

### Background calculation

We make observations of the background distributions of Tc scores between microenvironments from different sites. We compile a dataset of 1160 sites from a non-redundant set of 3D structures in PDB using these filters: (1) structures were solved using X-ray diffraction at resolutions higher than 2.0 Angstrom; (2) no two structures have greater than 40% sequence identity; (3) specifically bound small molecule ligands have more than the heavy atoms. The binding residues are defined as those having any atom within 6 Angstroms of the ligand molecules, resulting in a total of 22008 microenvironments. There are 242 possible types of pairs between 22 types of microenvironments centered on 20 residues types (two centers for residue W and Y), but not all of these are likely to be matched. For computational efficiency, we group residues by physical properties in order to avoid comparisons that are unlikely to yield high similarity scores. The comparisons are limited to pairs of microenvironments that fall within the same groups: positively charged (R H K), negatively charged (D E), polar (S T Q N W1 Y1), non-polar (A C G I L M P V) and aromatic (W2 Y2 F). This produces 72 microenvironment-pairs (out of the 200 possible) that we check for high similarity scores. Given each microenvironment-pair, we derive Tc scores from the above dataset and fit these score into normal distribution.

### Comparing two binding sites

Given the FEATURE microenvironments from two binding sites, we exhaustively calculate the raw Tc scores of all permissible microenvironment-pairs. We then normalize the Tc scores using the background frequency [34]:
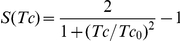



Tc_0_ is the value at which S(Tc) is zero.

In practice, Tc_0_ is the Tc score at the mode on the fitted cumulative distribution function (CDF) of a given type of microenvironment-pair (See Background calculation). The normalized value, S(Tc), measures the similarity between two microenvironments and is thus the **microenvironment similarity score**.

We search for the mutual best-scoring microenvironment-pairs between two binding sites and assign alignment to such pairs using an cutoff of S(Tc) less than −0.3. For example, between site A (microenvironments A1, A2, A3, A4 and A5) and site B (microenvironments B1, B2, B3, B4 and B5), we align A1 to B1 only when (1) S(Tc) between A1 and B1 is smaller than those between A1 and B1, B2, B4 B5, also smaller than those between B1 and A2, A3, A4, A5; (2) S(Tc) between A1 and B1 is smaller than −0.3. The sum of all aligned microenvironment-pairs is the overall similarity score between two binding sites, and is termed the binding site similarity score. We can vary the cutoff for S(Tc) to change the precision and resolution of the comparison.

### Benchmark datasets

We perform two sets of benchmark. The first benchmark identifies pairs of proteins that bind adenine-containing ligands, using two datasets [6] provided by Bourne group from USCD. Dataset I consists of 247 sites from non-redundant protein structures known to bind an adenine-containing ligand (ATP, ADP, NAD, FAD, SAH and SAM); Dataset II consists of 101 cavities from non-redundant protein structures believed not to bind an adenine-containing moiety. From Dataset I, we have 30381 pairs of sites that both bind adenine-containing ligands. Between Dataset I and II, we have 24947 control pairs of sites in which one site binds adenine-containing ligands and the other does not.

The second benchmark recognizes FAD binding sites from a large-scale dataset. A dataset consisting of 6958 druggable binding sites (Dataset-6958) was derived from scPDB entries [35] by filtering out PDB entries where atom coordinates of binding residues were incomplete. The binding sites were defined by including all the protein residues with at least one atom within 6 Angstrom of any ligand atom. Dataset-6958 includes a total of 249 FAD-binding proteins.

### Predicting inhibitor-binding profiles of kinases

We derive a subset of 984 binding sites from Dataset-6958, including proteins classified in eight different EC families: EC 2.7.1, EC 2.7.2, EC 2.7.3, EC 2.7.4, EC 2.7.10, EC 2.7.11, EC 2.7.12 and EC 2.7.13. A total of 203 sites bind to ATP, ANP or ADP. The other 781 structures bind to 633 other ligands.

Using the 203 sites involved in binding ATP, ANP or ADP, we generate pairs of binding sites from functionally and structurally distant kinases by applying two rules: (1) two kinases are from different EC sub-subgroups; (2) the identity of structural alignment by MAMMOTH [36] between the two kinases is not higher than 30%. This results in 6058 pairs of sites that bind to ATP, ANP or ADP.

We perform validation using experimental data from CHEMBL [28] and Ambit panel [29]. Ambit panel contains binding assays between 37 inhibitors (21 tyrosine kinase inhibitors, 15 serine-threonine kinase inhibitors, and 1 lipid kinase inhibitor), which are classified according to the targets for which they were originally developed, and 317 human kinases (287 different human protein kinases, three lipid kinases and 27 disease-relevant mutant variants). Staurosporine is a non-selective kinase inhibitor and is therefore removed from the validation dataset.

We use two standards to identify candidate inhibitors from Ambit panel: a stringent cutoff of Kd<1 uM and a ratio of off-target to primary target affinities (Kd off-target/Kd primary target) <100. Our local database downloaded from CHEMBL includes binding assays between 3199 protein domains and 541,137 compounds. It is a collection of assays from a variety of experimental studies and therefore the standards applied to Ambit Panel are not applicable to CHEMBL data. We first filter out data with confidence level less than seven. We then use either (1) a cutoff of IC50 less than 1 uM or (2) a cutoff of Kd less than 1 uM or (3) inhibition level higher than 90% at 1 uM as an indication of inhibition.

From the 6058 pairs of divergent kinases, we first search for pairs for which experimental data, from CHEMBL or Ambit, are available for both kinases. Given such a pair, if at least one compound/inhibitor satisfies the standards above for both kinases, the pair shares ligand binding and is a “positive-pair”; otherwise this pair is a “negative-pair”.

## Supporting Information

Figure S1 Performance of PocketFEATURE for predicting FAD-binding sites using different queries.
(PDF)

Figure S2 Performance of PocketFEATURE for predicting steroids binding sites.
(PDF)

Figure S3 Hierarchical clustering of 40 sites by binding site similarity scores calculated by PocketFEATURE.
(PDF)

Figure S4 Incorporating ligand chemoinformatics similarities in PocketFEATURE leads to prediction of higher confidence.
(PDF)

Table S1 Highly ranked off-target predictions.
(PDF)

Table S2 FEATURE property list.
(PDF)

Table S3 Functional centers of 22 types microenvironments.
(PDF)
