# Supplementary material for: Using Multiple Microenvironments to Find Similar Ligand-Binding Sites: Application to Kinase Inhibitor Binding
Source: PLoS Comput Biol. 2011 Dec 29;7(12):e1002326. doi: 10.1371/journal.pcbi.1002326 (PMC3248393; doi:10.1371/journal.pcbi.1002326)

Figure S1. Performance of PocketFEATURE for predicting FAD-binding sites using different queries

The two query sites are 1nhp that binds to an elongated FAD and 1fdr that binds to a bent butterfly FAD. PocketFEATURE is able to identify FAD-binding sites by using a query site that bind to a ligand in either an elongated or a bent butterfly shape.

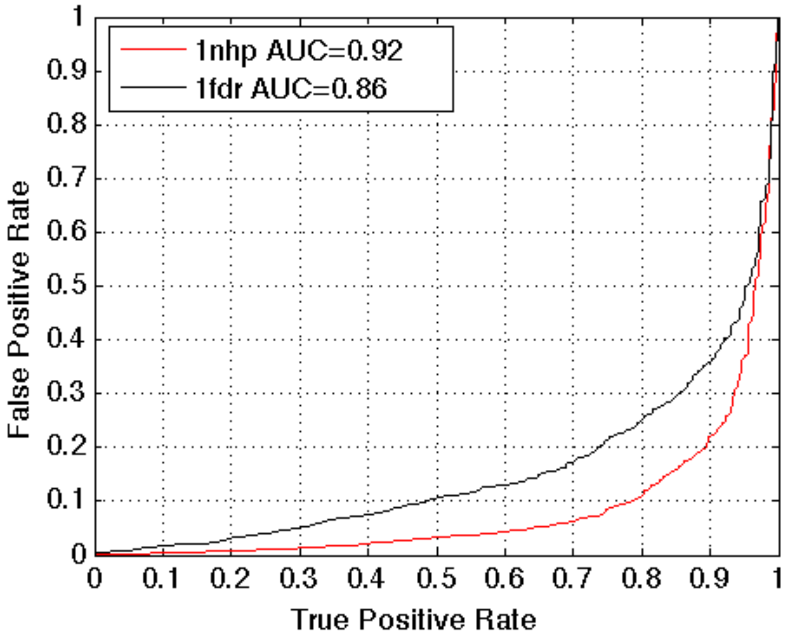

Supplement: Figure S1 — Performance of PocketFEATURE for predicting FAD-binding sites using different queries. (PDF) [file pcbi.1002326.s001.pdf]
