# Supplementary material for: Using Multiple Microenvironments to Find Similar Ligand-Binding Sites: Application to Kinase Inhibitor Binding
Source: PLoS Comput Biol. 2011 Dec 29;7(12):e1002326. doi: 10.1371/journal.pcbi.1002326 (PMC3248393; doi:10.1371/journal.pcbi.1002326)

Figure S2. Performance of PocketFEATURE for predicting steroids binding sites
The chemical structures of five hormone steroids progesterone, testosterone, estradiol, dihydrotesterone and equilenin are similar. In PDB, there are only 83 3D structures bound with these steroids. The numbers of non-hydrogen atoms of the five steroids range from 20 to 23.
We use a typical steroid binding site (1A28 bound with Progesterone) to detect all other steroid-binding sites from a large number of sites (Dataset-6985, see method section). The overall AUC is 0.826. It suggests the PocketFEATURE is able identify site similarity for non-adenine ligand. The size of steroids is smaller than that of ATP (31 non-hydrogen atoms) or FAD (53 non-hydrogen atoms). The performance is slightly low than that of using FAD as a query (Figure 3).
To further investigate the dependence on ligand sizes, we used two filters to generate two subsets from Dataset-6985. Filter-30 removes ligands with more than 30 non-hydrogen atoms and Filter-40 removes those with more than 40. PocketFEATURE achieve similar performance on the three datasets, suggesting the scoring and alignment process is robust.

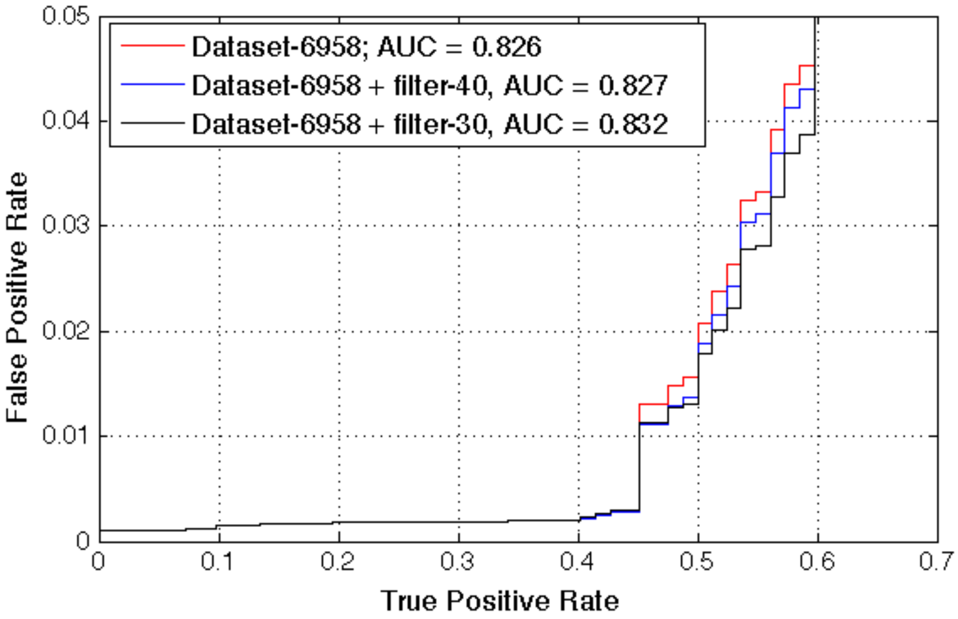

Supplement: Figure S2 — Performance of PocketFEATURE for predicting steroids binding sites. (PDF) [file pcbi.1002326.s002.pdf]
