# Supplementary material for: Using Multiple Microenvironments to Find Similar Ligand-Binding Sites: Application to Kinase Inhibitor Binding
Source: PLoS Comput Biol. 2011 Dec 29;7(12):e1002326. doi: 10.1371/journal.pcbi.1002326 (PMC3248393; doi:10.1371/journal.pcbi.1002326)
Supplement: Figure S3 — Hierarchical clustering of 40 sites by binding site similarity scores calculated by PocketFEATURE. (PDF) [file pcbi.1002326.s003.pdf]

Figure S3. Hierarchical clustering of 40 sites by binding site similarity scores calculated by PocketFEATURE

The ligand type is given in the capital letters (NAD, ATP, HEM stands for heme and STR stands for steroids). The PDB codes of the target protein structures are given in lower case letters. The 40 binding sites are derived from [1]. NAD and heme binding sites cluster quite well; ATP and STR binding sites show a very low level of separation.

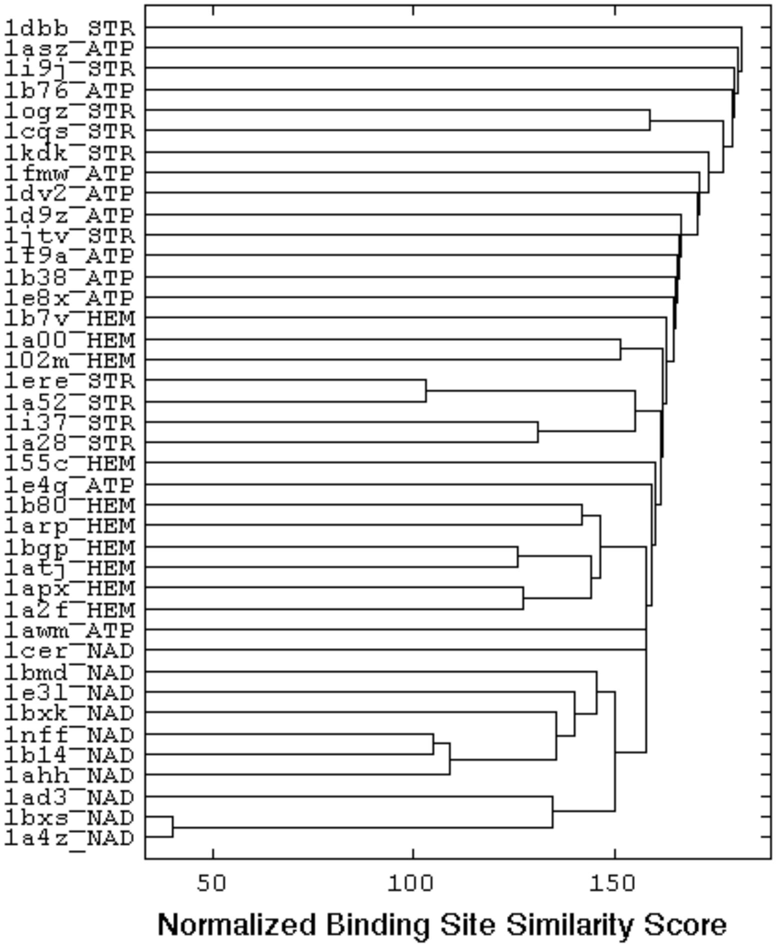

1. Morris RJ, Najmanovich RJ, Kahraman A, Thornton JM (2005) Real spherical harmonic expansion coefficients as 3D shape descriptors for protein binding pocket and ligand comparisons. Bioinformatics 21: 2347-2355.
