# Supplementary material for: Using Multiple Microenvironments to Find Similar Ligand-Binding Sites: Application to Kinase Inhibitor Binding
Source: PLoS Comput Biol. 2011 Dec 29;7(12):e1002326. doi: 10.1371/journal.pcbi.1002326 (PMC3248393; doi:10.1371/journal.pcbi.1002326)
Supplement: Figure S4 — Incorporating ligand chemoinformatics similarities in PocketFEATURE leads to prediction of higher confidence. (PDF) [file pcbi.1002326.s004.pdf]

Figure S4. Incorporating ligand chemoinformatics similarities in PocketFEATURE leads to prediction of higher confidence.

Procaspase-3 is involved in the activation cascade of caspases responsible for apoptosis execution. Procaspase activators bypass the normal upstream proapoptotic signaling cascades and induce rapid apoptosis in a variety of cell lines. Current research focuses on the discovery of small-molecule activators of procaspases [2]. Some activators are reported to enable direct control of caspases in apoptosis and in cell differentiation. We apply PocketFEATURE to identify binding sites of known drugs that are similar with the activator-binding sites in procaspases-3 with an aim to repurpose known small-molecule drugs. SEA calculates similarity between two target proteins based on chemoinformatics similarities between two sets of ligands that are known to bind the two sites. SEA similarity scores have been made available between procaspase-3 and 49 proteins with known drugs. A default cutoff of SEA is 1e-10. We applied PocketFEATURE to calculate binding site similarity scores between procaspase-3 and the 49 proteins. In PDB, there are 31 structures of high quality (x-ray resolution higher than 2.5 Angstrom) that bind to different ligand molecules. To determine a cutoff in binding site similarity scores, we first compared the 31 structures against a large druggable dataset and calculated the distributions of similarity scores. The cutoff for binding site similarity scores by PocketFEATURE is -1.5 (p-value cutoff =0.01). We then plotted binding site similarity scores between procaspase-3 and the 49 proteins against the corresponding SEA scores. The two measurements show a low level of correlation. It suggests that combining information from ligand molecules and target proteins result in stronger signals and predictions of higher confidence.

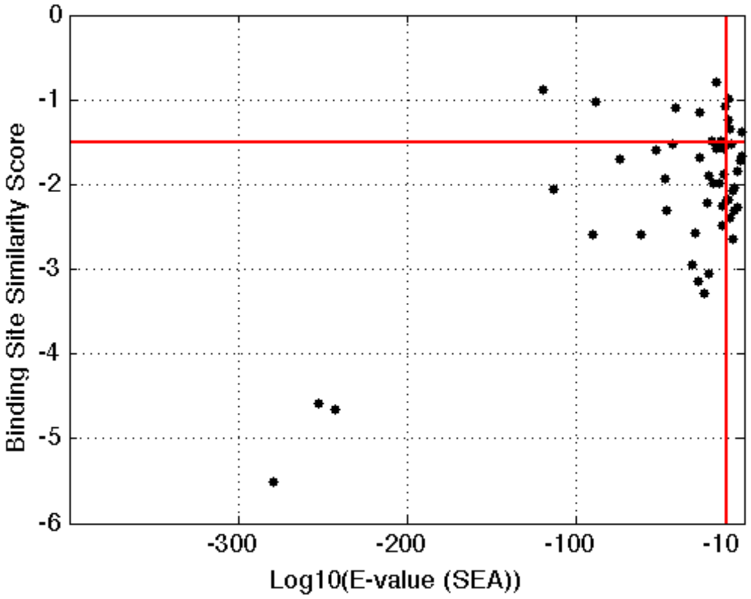


2. Wolan DW, Zorn JA, Gray DC, Wells JA (2009) Small-molecule activators of a proenzyme. Science 326: 853-858.
