# Supplementary material for: Using Multiple Microenvironments to Find Similar Ligand-Binding Sites: Application to Kinase Inhibitor Binding
Source: PLoS Comput Biol. 2011 Dec 29;7(12):e1002326. doi: 10.1371/journal.pcbi.1002326 (PMC3248393; doi:10.1371/journal.pcbi.1002326)
Supplement: Table S1 — Highly ranked off-target predictions. (PDF) [file pcbi.1002326.s005.pdf]

Table S1. Highly ranked off-target predictions
We applied PocketFEATURE to predicted off-targets for CETP inhibitors (specifically Torcetrapib) from a non-redundant subset of PDB for 1200 human proteins. Most of PocketFEATURE predictions are involved in lipid transfer or binding and signal transduction pathway. One group published a panel of 20 off-targets for Torcetrapib predicted by SOIPPA. SOIPPA predictions have been refined and validated by docking methods and critical human curation. Between the 20 SOIPPA off-targets by SOIPPA and the 34 PocketFEATURE predictions, seven are the same (highlight in grey). The hyper-geometric *p-value* for enrichment is 1e-4.

PDB	Chain	Ligand ID	Protein name
1kkq	A	471	peroxisome proliferator activated receptor
1y0s	A	331	peroxisome proliferator activated receptor delta
2p54	A	735	peroxisome proliferator-activated receptor alpha
2a1l	A	PCW	phosphatidylinositol transfer protein beta isoform
3cqv	A	HEM	nuclear receptors REV-ERB alpha
1yuc	B	EPH	orphan nuclear receptor nr5a2
1nrl	B	SRL	orphan nuclear receptor pxr
1yow	A	P0E	steroidogenic factor 1
1lqv	B	PTY	endothelial protein c receptor
1nhz	A	486	glucocorticoid receptor
2oo8	X	RAJ	angiopoietin-1 receptor
1dkf	B	BMS	retinoic acid receptor-alpha
1onq	A	SLF	t-cell surface glycoprotein cd1a
1gzp	A	GM2	t-cell surface glycoprotein cd1b
3huj	A	AGH	t-cell surface glycoprotein cd1d
1jnk	A	ANP	c-jun n-terminal kinase
3eqc	A	AGS	dual specificity mitogen-activated protein kinase kinase 1
3gp0	A	NIL	mitogen-activated protein kinase 11
2clq	B	STU	mitogen-activated protein kinase kinase kinase 5
3efw	A	AK8	serine/threonine-protein kinase 6
3fxz	A	FLL	serine/threonine-protein kinase PAK1
1tbf	A	VIA	cgmp-specific 3',5'-cyclic phosphodiesterase
3bjc	A	WAN	cgmp-specific 3',5'-cyclic phosphodiesterase
1uu3	A	LY4	3-phosphoinositide dependent protein kinase-1
2bzg	A	SAH	thiopurine s-methyltransferase
3ckk	A	SAM	trna (guanine-n(7)-)-methyltransferase
2ozu	A	ACO	histone acetyltransferase myst3
2p0w	A	ACO	histone acetyltransferase type b catalytic subunit
1k3y	A	GTX	glutathione s-transferase A1
2vcv	I	ASD	glutathione s-transferase A3
1ewf	A	PC1	bactericidal/permeability-increasing protein
2ag4	A	LP3	ganglioside gm2 activator
2cvd	D	HQL	glutathione-requiring prostaglandin d synthase
3kh6	A	APR	polymerase 15
1r5l	A	VIV	alpha-tocopherol transfer protein
1qip	B	GNB	lactoylglutathione lyase
