# Supplementary material for: Using Multiple Microenvironments to Find Similar Ligand-Binding Sites: Application to Kinase Inhibitor Binding
Source: PLoS Comput Biol. 2011 Dec 29;7(12):e1002326. doi: 10.1371/journal.pcbi.1002326 (PMC3248393; doi:10.1371/journal.pcbi.1002326)
Supplement: Table S2 — FEATURE property list. (PDF) [file pcbi.1002326.s006.pdf]

Table S2: FEATURE property list

ATOM-TYPE-IS-C	RESIDUE_NAME_IS_GLU
ATOM-TYPE-IS-CT	RESIDUE_NAME_IS_GLY
ATOM-TYPE-IS-Ca	RESIDUE_NAME_IS_HIS
ATOM-TYPE-IS-N	RESIDUE_NAME_IS_ILE
ATOM-TYPE-IS-N2	RESIDUE_NAME_IS_LEU
ATOM-TYPE-IS-N3	RESIDUE_NAME_IS_LYS
ATOM-TYPE-IS-Na	RESIDUE_NAME_IS_MET
ATOM-TYPE-IS-O	RESIDUE_NAME_IS_PHE
ATOM-TYPE-IS-O2	RESIDUE_NAME_IS_PRO
ATOM-TYPE-IS-OH	RESIDUE_NAME_IS_SER
ATOM-TYPE-IS-S	RESIDUE_NAME_IS_THR
ATOM-TYPE-IS-SH	RESIDUE_NAME_IS_TRP
ATOM-TYPE-IS-OTHER	RESIDUE_NAME_IS_TYR
PARTIAL-CHARGE	RESIDUE_NAME_IS_VAL
ATOM-NAME-IS-ANY	RESIDUE_NAME_IS_HOH
ATOM-NAME-IS-C	RESIDUE_NAME_IS_OTHER
ATOM-NAME-IS-N	RESIDUE_CLASS1_IS_HYDROPHOBIC
ATOM-NAME-IS-O	RESIDUE_CLASS1_IS_CHARGED
ATOM-NAME-IS-S	RESIDUE_CLASS1_IS_POLAR
ATOM-NAME-IS-OTHER	RESIDUE_CLASS1_IS_UNKNOWN
HYDROXYL	RESIDUE_CLASS2_IS_NONPOLAR
AMIDE	RESIDUE_CLASS2_IS_POLAR
AMINE	RESIDUE_CLASS2_IS_BASIC
CARBONYL	RESIDUE_CLASS2_IS_ACIDIC
RING-SYSTEM	RESIDUE_CLASS2_IS_UNKNOWN
PEPTIDE	SECONDARY_STRUCTURE1_IS_3HELIX
VDW-VOLUME	SECONDARY_STRUCTURE1_IS_4HELIX
CHARGE	SECONDARY_STRUCTURE1_IS_5HELIX
NEG-CHARGE	SECONDARY_STRUCTURE1_IS_BRIDGE
POS-CHARGE	SECONDARY_STRUCTURE1_IS_STRAND
CHARGE-WITH-HIS	SECONDARY_STRUCTURE1_IS_TURN
HYDROPHOBICITY	SECONDARY_STRUCTURE1_IS_BEND
MOBILITY	SECONDARY_STRUCTURE1_IS_COIL
SOLVENT-ACCESSIBILITY	SECONDARY_STRUCTURE1_IS_HET
RESIDUE_NAME_IS_ALA	SECONDARY_STRUCTURE1_IS_UNKNOWN
RESIDUE_NAME_IS_ARG	SECONDARY_STRUCTURE2_IS_HELIX
RESIDUE_NAME_IS_ASN	SECONDARY_STRUCTURE2_IS_BETA
RESIDUE_NAME_IS_ASP	SECONDARY_STRUCTURE2_IS_COIL
RESIDUE_NAME_IS_CYS	SECONDARY_STRUCTURE2_IS_HET
RESIDUE_NAME_IS_GLN	SECONDARY_STRUCTURE2_IS_UNKNOWN
