# Supplementary material for: Using Multiple Microenvironments to Find Similar Ligand-Binding Sites: Application to Kinase Inhibitor Binding
Source: PLoS Comput Biol. 2011 Dec 29;7(12):e1002326. doi: 10.1371/journal.pcbi.1002326 (PMC3248393; doi:10.1371/journal.pcbi.1002326)
Supplement: Table S3 — Functional centers of 22 types microenvironments. (PDF) [file pcbi.1002326.s007.pdf]

Table S3. Functional centers of 22 types microenvironments

Non-polar		Polar		Positive-charged	
G	Hypothetical CB	S	OG	H	NE2 ND
C	SG	T	OG1	K	NZ
A	CB	Q	OE1 CD NE2	R	CZ
I	CB	N	OD1 CG ND2		
L	CB	W1	NE1	Aromatic	
V	CB	Y1	OH	W2	CD2 CE2 CE3 CZ2 CZ3 CH2
P	N CA CB CD CG	Negative charged		Y2	CG CD1 CD2 CE1 CE2 CZ
M	SD	D	OD1 CG OD2	F	CG CD1 CD2 CE1 CE2 CZ
		E	OE1 CD OE2		
